# Acknowledgment of Reviewers

**DOI:** 10.1093/geroni/igae001

**Published:** 2024-02-14

Scientific progress depends on the generosity of reviewers who assist editors by sharing their time and expertise in the peer review process. Steven M. Albert, PhD, FGSA, Editor-in-Chief, Jennifer Tehan Stanley, PhD, FGSA, Deputy Editor-in-Chief, and the Associate Editors of *Innovation in Aging* wish to thank the following individuals for their assistance in reviewing manuscripts during 2023.

Abrahamson, Kathleen

Adams, Kathryn

Adedeji, Adekunle

Ajibo, Henry

Ajrouch, Kristine

Akinrolie, Olayinka

Aldwin, Carolyn

Allen, Julie

Angel, Jacqueline

Aroonsrimorakot, Sayam

Ashida, Sato

Aung, Myo Nyein

Austin, Charles

Au-Yeung, Wan-Tai

Awuviry-Newton, Kofi

Ayalon, Liat

Baker, Tamara

Balachandran, Arun

Barry, Lisa

Beach, Brian

Beach, Scott

Bellelli, Giuseppe

Benge, Jared

Bergen, Gwendolyn

Berkowsky, Ronald

Blumen, Helena

Boltz, Marie

Boot, Walter

Bowers, Barbara

Brewster, Katharine

Bristol, Alycia

Britt-Spells, Angelitta

Brooks, Amber

Brown, Lauren

Bucy, Taylor

Burnes, David

Burns, Shane

Buta, Brian

Calkins, Margaret

Camacho, David

Campbell, Anthony

Carnahan, Jennifer

Carr, Deborah

Cawthon, Peggy

Chakraborty, Rishika

Chang, E-Shien

Chang, Haeyoon

Chang, Pei-Fen

Charlesworth, Georgina

Chatterjee, Sudeshna

Chaudhury, Habib

Chen, Gong

Chen, Ke

Chen, Shanquan

Chen, Xiayu

Chippendale, Tracy

Chopik, William

Chou, Li-Wei

Choudhury, Renoa

Christie, Julie

Chu, Li

Chukwuorji, JohnBosco

Chung, Ren-Hua

Cichy, Kelly

Clark, Nancy

Cloud, David

Cochrane, Barbara

Cohen, Aaron

Cohn-Schwartz, Ella

Coleman, Carissa

Con, Gulcin

Connell, Cathleen

Conwell, Yeates

Cooke, Heather

Crittenden, Jennifer

Crow Brauer, Megan

Cunha, Vanessa

Cutchin, Malcolm

Dansereau, Lisette

Dassel, Kara Bottiggi

Davey, Adam

Davidson, Patrick

Davila, Heather

De Liema, Marguerite

de Medeiros, Kate

Di Gessa, Giorgio

Dobash, Dawson

Dong, Fanghong

Dunlap, Pamela

Ehrenkranz, Rebecca

Enders-Slegers, Marie-José

Engel, Rafael

Erickson, Alexander J.

Esiaka, Darlingtina

Eze, John E.

Farsijani, Samaneh

Fennell, Gillian

Fingerman, Karen

Finkel, Deborah

Fitzgerald, Michael

Flatt, Jason

Francis Essien, Nsidibe

Frey, Katelyn

Fu, Xinxing

Fuller, Heather

Galik, Elizabeth

Gao, Xu

Garcia, Marc

Gately, Megan

Ge, Ning

Ge, Shaoqing

Gilligan, Megan

Glicksman, Allen

Gómez-Olivé, F. Xavier

Gripko, Monica

Grol-Prokopczyk, Hanna

Hager, Erricka

Haghighi, Paniz

Haighton, Catherine

Hall, Latoya

Hamann, Darla

Han, Claire

Han, Sae Hwang

Hancock, David

Handler, Steven

Hanes, Douglas

Harrington, Erin

He, Dongsheng

Heid, Allison

Helzner, Elizabeth

Hermer, Linda

Hetherington-Rauth, Megan

Holmes, Sarah

Horgas, Ann

Hou, Bo

Hsu, Hui-Chuan

Hu, Kai

Hu, Rita

Huang, Derong

Huang, Gaojian

Huang, Tim

Hubner, Sarah

Huseth-Zosel, Andrea

Hutchins, Franya

Idler, Ellen

Idowu, Kareem

Ie, Kenya

Ihara, Emily

Ilevbare, Femi

Issahaku, Paul

Jackson, Yolanda

Jacobson, Mireille

Jain, Urvashi

Jansen, Carl-Philipp

Jiang, Fan

Johnson, Alisa

Johnson, Mary Ann

Jolliff, Anna

Joseph, Anjali

Judge, Katherine

Jung, Daniel

Kalu, Michael

Kaut, Kevin

Kessler, Eva-Marie

Khan, Maham

Kindratt, Tiffany

Kittle, Krystal

Klinedinst, Tara

Koh, Vanessa

Kohler, Iliana

Kolk, Daisy

Kowal, Paul

Kritz, Marlene

Kuo, TsuAnn

Kurth, Maria

Lai, Daniel

Lanthier-Labonte

Lee, Chungsup

Lee, Sangil

Lee, Yeonjung

Leedahl, Skye

Lehning, Amanda

Levy, Sheri

Levy-Storms, Lene

Li, Guannan

Lifshitz, Hefziba

Lin, Feng

Lin, Hongmei

Lin, Jielu

Lin, Xin Yao

Lin, Zhiyong

Litwin, Howard

Liu, Carol

Liu, Darren

Liu, Leilei

Liu, Xinran

Liu, Yujun

Lloyd-Sherlock, Peter

Loomer, Lacey

López-Ortega, Mariana

Loughnane, Megan

Luo, Ye

Ma, Chenxinan

Manalel, Jasmine

Mancuso, Carol

Manthorpe, Jill

Marques, Sibila

Martire, Lynn

Matamela, Nyambeni

Matias-Guiu, Jordi

Mattos, Meghan

Mbuthia, Kezia

McDougall Jr., Graham

McGuire, Lisa

McInnes, Lynn

Meng, Hongdao

Meyer, Kylie

Miller, Lisa

Minyo, Morgan

Mitzner, Tracy

Miyawaki, Christina

Mondro, Anne

Monin, Joan

Moody, Harry R.

Morgan, Erin

Morris, Ashley

Morrow, Daniel

Morrow-Howell, Nancy

Mu, Christina

Mussie, Kirubel

Mwangi, Samuel

Nah, Suyoung

Nakagawa, Takeshi

Nakagomi, Atsushi

Naugle, Kelly

Neller, Sarah

Ng, Reuben

Nicklett, Emily

Niznik, Joshua

Nkwata, Allan

Noguchi, Taiji

Nwakasi, Candidus

O’Brien, Myles

O’Connor, Deborah

Odeyemi, Emmanuel

Oedingen, Carina

Ogletree, Aaron

Oh, Patricia

Okafor, Chiedozie

Olawa, Babatola

Ong, Anthony D.

Onu, Desmond

O’Rourke, Norm

Ory, Marcia

Oswald, Frank

Otsuki, Takuya

Palmer, Catherine

Pan, Thet Htoo

Peng, Yisheng

Permanyer, Inaki

Peterson, Colleen

Peterson, Lindsay

Phongtankuel, Vee

Pickering, Carolyn

Pritchard, Kevin

Puga, Frank

Puurveen, Gloria

Qi, Xiang

Qiao, Yujia (Susanna)

Qiu, Mengying

Rabins, Peter

Raj, Minakshi

Ralston, Margaret

Rand, Stacey

Ravyts, Scott

Rebello, Candida

Reed, Nicholas

Reniers, Peter

Reyes, Adriana

Reynolds, Charles

Rhodus, Elizabeth

Richards, Marcus

Riffin, Catherine

Ritchie, Christine

Roberto, Karen

Rockette-Wagner, Bonny

Roes, Martina

Rosso, Andrea

Roy, Moushumi

Roydhouse, Jessica

Ryan, Lindsay

Sakamoto, Mariko

Samper Ternent, Rafael

Sands, Laura

Santilli, Josie

Scheibl, Fiona

Schneider, Stefan

Schulz, Richard

Schumacher, Benjamin

Seckin, Gul

Selvam, Sahaya

Sharma, Anjali

Shaw, Benjamin

Shelley, Mack

Shin, Esther

Simpson, Vicki

Siu, Ka-Chun

Sivertsson, Fredrik

Speechley, Mark

Stahl, Sarah

Stasiulis, Elaine

Strupp, Julia

Su, Tai-Te

Sun, Fei

Taeckens, Ashley

Tang, Fengyan

Taylor, Harry

Taylor, Robert

Teresi, Jeanne

Thomas, Patricia

Thuku, Pauline

Toto, Pamela

Toyoshima, Aya

Tramujas Vasconcellos Neumann, Lycia

Trevisan, Caterina

Tripathi, Arpita

Turner, Jennifer

Turner, Shelbie

Ugueto-Rey, Gustavo

Ugwu, Lawrence

Ugwu, Lawrence Ejike

Van Den Bossche, Maarten

Van den Bulcke, Laura

Vilaplana-Prieto, Cristina

Vo, Thang

Wagner, Laura

Wallhagen, Margaret I.

Wang, Helen

Wang, Jing

Wang, Jiwen

Wang, Kaipeng

Ward, Richard

Warner, David

Welburn, Sharon

White, Kellee

Wiemers, Emily

Wilkinson, Lindsay

Winett, Richard

Witham, Miles

Wong, Stephanie

Wu, Chenkai

Wu, Xinyin

Wu, Yijin

Xu, Beibei

Xu, Jiayuan

Yang, Yulin

Yao, Yao

Yazawa, Aki

Ye, Minzhi

Yorgason, Jeremy

Yow, W. Quin

Yu, Bin

Zaheed, Afsara

Zanjani, Faika

Zhang, Li

Zhang, Yalu

Zhang, Yongkang

Zhang, Yuan

Zhou, Yuanjin

Zhu, Huoyun

Zurlo, Karen

